# Preoperative percutaneous Onyx embolization of carotid body paragangliomas with balloon test occlusion

**DOI:** 10.3389/fneur.2023.1132100

**Published:** 2023-04-14

**Authors:** Martina Kelblová, Jiří Vaníček, Břetislav Gál, Jan Rottenberg, Martin Bulik, Petra Cimflová, Tomáš Křivka

**Affiliations:** ^1^Department of Medical Imaging, St. Anne’s University Hospital and Faculty of Medicine, Masaryk University, Brno, Czechia; ^2^Department of Otorhinolaryngology and Head and Neck Surgery, St. Anne’s University Hospital and Faculty of Medicine, Masaryk University, Brno, Czechia

**Keywords:** carotid body tumor, paraganglioma, percutaneous embolization, Onyx, balloon test occlusion

## Abstract

**Objectives:**

The study aims to analyze our first experience with direct percutaneous embolization of carotid body tumors (CBTs) using ethylene-vinyl alcohol copolymer (Onyx) along with balloon test occlusion (BTO).

**Methods:**

A retrospective preliminary single-center study was conducted at the Otorhinolaryngology and Head and Neck Surgery Department and the Medical Imaging Department of the University Teaching Hospital. A consecutive series of three patients with CBTs was treated at the local institution between October 2018 and June 2019. All three patients underwent preoperative percutaneous embolization using ethylene-vinyl alcohol copolymer (Onyx 18) with the addition of BTO. Outcome measures were the percentage of tumor devascularization, intraoperative blood losses, and operation times. BTO was evaluated by clinical neurological examination and neurosonological transcranial Doppler examination of the middle cerebral artery (MCA).

**Results:**

Devascularization of all three tumors was complete or near complete. All three tumors were surgically extirpated with excellent surgical outcomes. The blood losses were minimal, and the average operation time was 2 h and 8 min. BTO was positive in one patient, which was valuable additional information on carotid branches ligation limitations. The other two patients showed negative BTOs with the result of safety of eventual carotid arteries ligations.

**Conclusion:**

Preoperative direct percutaneous embolization of CBT with Onyx is a highly effective procedure that significantly facilitates surgery. BTO provides valuable additional information on the most appropriate and safe surgical approach.

## 1. Introduction

Carotid body paragangliomas or carotid body tumors (CBTs) are the most common head-and-neck paragangliomas (1, 2). They are rare, mostly benign, highly vascular neoplasms of the paraganglia. Their incidence is estimated at 1:30,000, accounting for 3% of all paragangliomas (2, 3). CBT typically presents as a slow-growing, painless lateral neck mass. In advanced stages, hoarseness or dysphagia may appear due to the involvement of the vagal nerve. Pulsatile tinnitus may also occur on the tumor side due to the high-flow state (2, 3). Imaging modalities for the CBT diagnosis include magnetic resonance angiography (MRA) or CT angiography (CTA). With the use of either modality, early enhancing soft-tissue tumor is characteristically seen at the level of the carotid bifurcation, splaying the internal and external carotid artery to create the “lyre sign.” Magnetic resonance imaging (MRI) will demonstrate a “salt and pepper” pattern within the tumor, representing the flow voids of its prominent blood vessels (2).

The treatment strategies include conservative management, radiotherapy, and surgical resection, the only curative for resectable CBTs (3). However, it has pitfalls, such as high vascularity and intimate relation of the vagal nerve and carotid adventitia. These facts bring a non-negligible risk of intraoperative bleeding, vagal nerve injury, and stroke (3). Careful preoperative planning and patient selection are essential for a successful surgical outcome. A landmark in the assessment of resectability is the surgical Shamblin classification of CBTs into three groups according to their relationship with the carotid vessels (4). Shamblin I tumors are minimally attached to the vessels, and easily resectable. Shamblin II tumors are partially surrounding vessels, and are more adherent to vessel adventitia. They are difficult to dissect but amenable to careful resection. Shamblin III tumors have an intimate adherent relationship to the entire circumference of the carotid bifurcation. Their resection requires ICA resection with vessel replacement (4, 5).

The value of preoperative embolization has been reported (6–13). Endovascular embolization facilitates surgery by reducing hypervascularity (6, 7). However, this treatment technique is limited due to tiny feeding branches from ECA and ICA that are not easily accessible for direct catheterization (8–10). Therefore, percutaneous embolization of the lesion by a direct puncture was proposed as a better solution (8–13). This study aims to report our first experience with direct percutaneous embolization of CBTs using Onyx besides BTO.

## 2. Materials and methods

A consecutive series of three patients with CBTs treated at the local institution between October 2018 and June 2019 with preoperative percutaneous embolization using ethylene-vinyl alcohol copolymer (Onyx 18) was retrospectively reviewed. The age of the patients ranged from 22 to 68, one was male, and two were female. The largest diameter of the tumor ranged from 3 to 4.5 cm. According to the Shamblin classification, two tumors were class I, and one was class II. All patients noted slowly growing palpable lateral neck mass; one experienced subtle pain. None of the patients demonstrated cranial nerve palsy.

**Figure 1:**
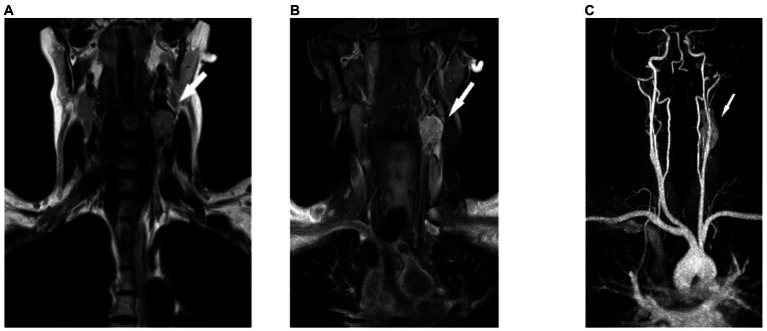
Carotid body tumor on the left side. **(A)** MR T2-weighted coronal image. **(B)** MR T1-weighted coronal image with fat suppression after contrast medium administration. **(C)** Contrast-enhanced MR angiography (CE-MRA), and maximum intensity projection (MIP) reconstruction in the coronal plane.

All patients underwent preoperative contrast-enhanced MRI (Figures 1A,B) and MRA imaging (Figure 1C), revealing a highly vascular expansion in the typical location.

Prior to the scheduled surgical extirpation, digital subtraction angiography (DSA) and percutaneous embolization were performed. Before the embolization, BTO was completed. The surgical resection was performed 3 to 4 days after the successful procedure to avoid temporary soft-tissue edema resulting from the aseptic inflammatory reaction to the embolization agent. The follow-up MRI and MRA were performed 3 to 4 months after the operation.

### 2.1. Radiointerventional procedures

All patients signed informed consent before the endovascular procedures.

#### 2.1.1. Balloon test occlusion

**Figure 2:**
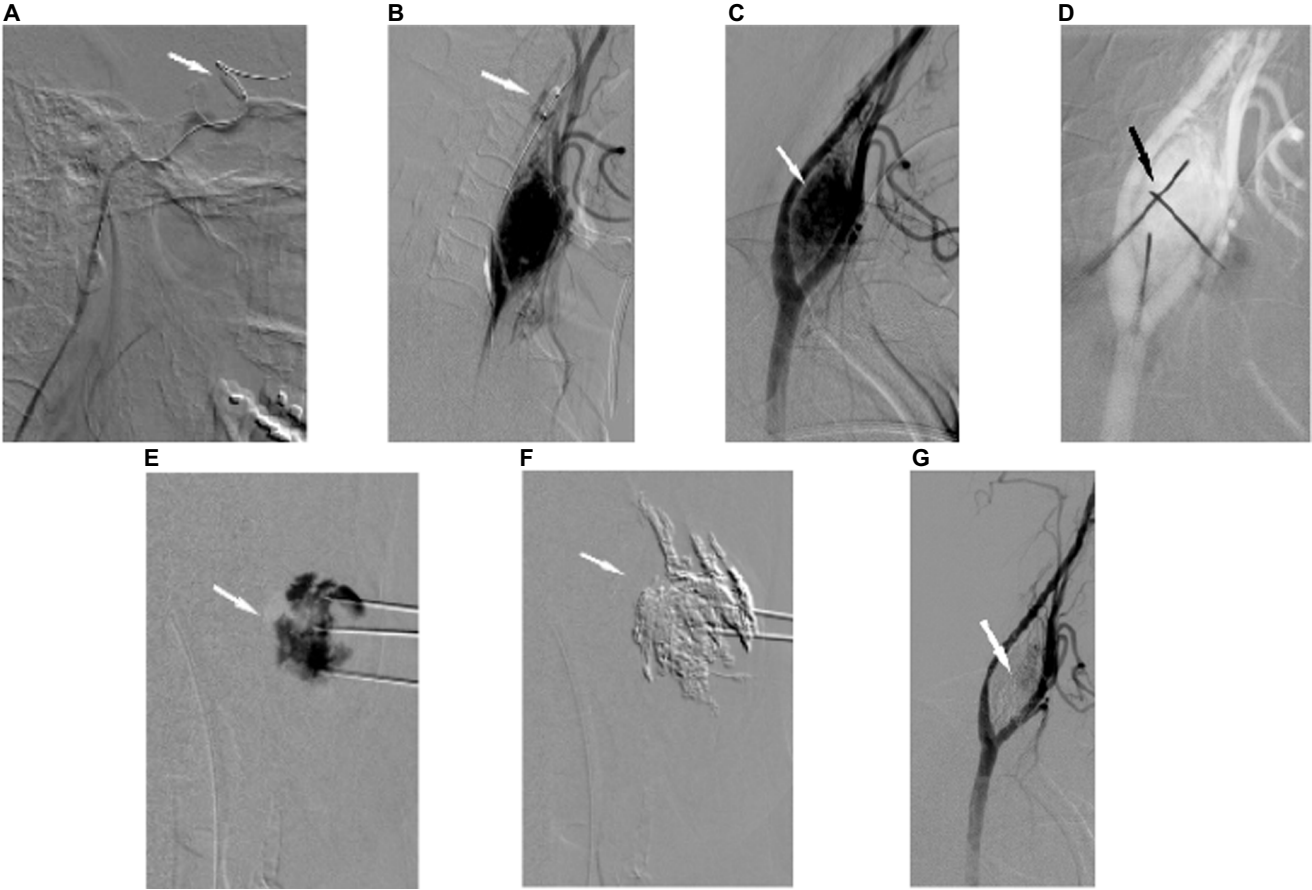
DSA of the left ICA with the **(A)** first balloon occlusion test (BOT): balloon occlusion of the supraophthalmic segment of the left ICA. **(B)** Second BOT: balloon occlusion of the cervical segment of the left ICA. **(C)** Carotid body tumor on the left side, DSA of the left common carotid artery (CCA); sagittal view. **(D)** Control of the positions of 3 percutaneously inducted Chiba needles into the tumor mass. **(E)** Parenchymogram of the CBT on the left side. **(F)** Fluoroscopic control of percutaneous injection of Onyx 18 into CBT through Chiba needles. **(G)** DSA of the left CCA after percutaneous injection of Onyx into the CBT mass with devascularization of the tumor.

The guiding catheter was introduced into the common carotid artery (CCA) on the tumor side (transfemoral approach), and a baseline carotid angiogram was obtained. In the first phase, Balloon test occlusion (BTO) was performed using an occlusive balloon (HyperForm), which provided temporary occlusion of ICA at the level of the supraophthalmic segment and subsequently at the extracranial ICA (Figures 2A,B).

#### 2.1.2. Percutaneous embolization of CBTs

The second phase of the radiointerventional procedure (percutaneous embolization) was performed under general anesthesia to avoid pain due to Onyx injection. In compliance with aseptic principles, we found an optimum overview of the target lesion and the adjacent ICA and ECA on a biplane angiography unit (Artis zee, Siemens, Erlangen, Germany). The percutaneous tumor puncture was then performed under the road-mapping technique with two CCA projections (Figure 2C). The continuous contrast medium administration was used during the puncture to confirm the optimal position of the needle tip within the larger vessel supplying the adequate portion of the tumor bed. Three to four Chiba needles were placed within the tumor mass (Figure 2D). Once the percutaneous approach was achieved, a particular tumor’s vascular territory was visualized with a parenchymogram (Figure 2E). After flushing a delivery system with dimethyl-sulfoxide (DMSO) to avoid precipitation of the embolic material within the delivery system, slow injection of 6% ethylene-vinyl alcohol copolymer (Onyx 18) was performed under continuous fluoroscopy (Figure 2F). The injection of Onyx was completed when the previously visualized portion of the tumor supplied by the given vascular territory was occluded. The percutaneous embolization was then repeated through another previously inserted Chiba needle until the complete/near-complete occlusion of the CBT was achieved. The grade of tumor devascularization was subsequently confirmed on the control CCA angiogram (Figure 2G).

### 2.2. Outcome measures

BTO parameters were clinical neurological symptoms and hemodynamically significant changes in the blood flow velocity of the middle cerebral artery (MCA), monitored by a neurosonologist in 25 min interval through clinical evaluation and transcranial Doppler sonography. Their occurrence was suggestive of a positive test result. In the opposite scenario, the test was considered negative. Two interventional radiologists evaluated the percentage of tumor devascularization by comparing pre-and post-embolization DSA images. The intraoperative blood losses and operational times were obtained from the surgery operative reports.

## 3. Results

The BTO showed the Willis’ circle insufficiency in one patient during the supraophthalmic ICA occlusion with a rapid onset of aphasia and a critical depression of the MCA flow velocity. A complete symptom recovery was achieved after immediate release of the temporary occlusion with an unremarkable subsequent ICA angiogram. The following occlusion of the extracranial ICA was performed without any clinical symptoms or substantial hemodynamic changes, suggesting sufficient collateral capacity *via* ECA through the ophthalmic artery.

BTOs were negative for intracranial and extracranial ICA segmental occlusions in the other two patients, which was considered safety proof for eventual intraoperative ligation of ICA or ECA.

All three percutaneous embolization procedures were completed with no intraprocedural complications. The CBTs’ devascularization percentages were 90%, 95%, and 100%. CBTs of all three patients were extirpated en block within 3 to 4 days after the embolization. The intraoperative blood losses were less than 150 mL in all three cases, and histopathological findings confirmed the carotid body tumor (CBT) with extensive content of embolic agent in vascular spaces.

In one case, ligation of the superior thyroid artery was required. No other ligation of carotid artery branches was necessary. Two patients demonstrated no neurological symptoms after the tumor extirpation, and one had transient ipsilateral vocal fold palsy due to a temporary vagal nerve lesion.

**Figure 3:**
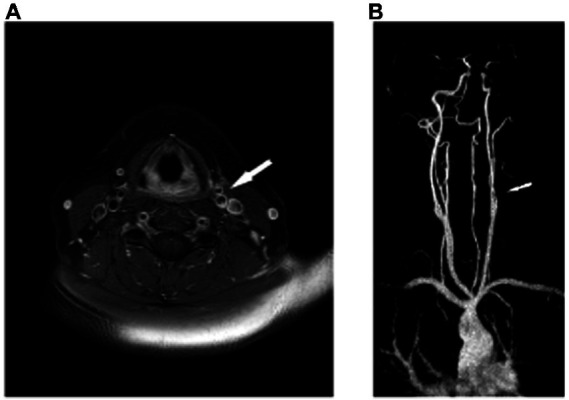
**(A)** Postoperative MR T1-weighted axial image of the neck with fat suppression after contrast medium administration at the level of CCA bifurcation with evidence of no mass. **(B)** Postoperative contrast contrast-enhanced MR angiography (CE-MRA) of the neck, maximum intensity projection (MIP) reconstruction in the coronal plane with evidence of no pathological vascularization. Only mild irregularities of CCA branches were demonstrated with no significant stenosis.

The follow-up MRI and MRA showed no residual tumor mass or pathological vascularization (Figures 3A,B).

The preoperative and postoperative characteristics of all patients are summarized in Table 1.

**Table 1:** Preoperative and postoperative characteristics of all patients.

Parameters	Patient 1	Patient 2	Patient 3
Sex (male/female)	Male	Female	Female
Age (years)	22	68	45
CBT tumor position	Right side	Left side	Left side
Maximal tumor size (cm)	4.5	4	3
Preoperative clinical symptoms	Palpable mass, little painful	Palpable mass, painless	Palpable mass, painless
Percentage of devascularization (%)	90	95	100
Operation duration (hours, min)	2 h 5 m	2 h 5 m	2 h 15 m
Intraoperative blood loss (mL)	Up to 150	Up to 150	Up to 150
Postoperative neurological deficit	No	Temporary vocal fold palsy	No

## 4. Discussion

Surgical resection is the preferable treatment for CBT (2, 3). Preoperative embolization reduces blood loss substantially (6–13). Other intraoperative risks, specifically neurological damage, proved a more complex, including factors such as tumor grade and surgical team experience (3–5).

Most CBT arterial feeders arise from ECA, usually from the ascending pharyngeal artery; others from CCA’s vasa vasorum or ICA (meningeal branch) (8). Although successful experience with transarterial embolization has been shown (6, 7), the complete tumor body embolization was not easily achievable *via* the transarterial approach as the tiny tumor vasculature was not easily accessible for highly selective or super-selective catheterization. The percutaneous embolization by direct puncture was suggested as a more suitable solution, allowing for the obliteration of the entire CBT tumor bed (8–13). Nevertheless, the complexity of the CBT vasculature requires multiple access points/punctures (commonly 3–4) even in the percutaneous approach to increase the operator’s chances to cover and embolize most of the tumor body.

As for the embolic agent type, we preferred the non-adhesive agent Onyx (11–13) to tissue glue cyanoacrylate (9, 10). Although no direct comparative studies for these two are available, Onyx has slower precipitation, enabling deeper penetration into the tumor bed and facilitating more controlled injection (11, 12, 14). As with any vascular embolization, there is a certain but relatively small risk of Onyx deviation into the parent artery. To minimize this risk, it is crucial to administer Onyx under fluoroscopic guidance and control its spread within the tumor bed.

We report the advantage of BTO in addition to the percutaneous embolization technique. It evaluates the collateral capacity of the Willis’ circle and/or ECA to supply intracranial ICA branches in the case of the necessity of intraoperatinal carotid arteries ligations due to intimate adhesion of the tumor.

Temporary occlusion of the supraophthalmic ICA segment evaluates the collateral capacity of the Willis’ circle to supply intracranial ICA branches beyond the point of occlusion. When the first test result is negative, eventual intraoperative ligation of both CCA branches (ICA and ECA) is considered safe. If it is positive, subsequent occlusion of the extracranial ICA segment follows to evaluate the collateral capacity of ECA through the ophthalmic artery. If this second test is negative, eventual intraoperative ligation of ICA is considered safe, but ECA must remain patent due to the additional collateral supply of intracranial ICA branches. In the case of a positive result of the second test, eventual ligation of ICA requires immediate vessel function replacement during operation. Otherwise, the risk of cerebral ischemia is unacceptably high. Finally, BTO with various modifications is a well-known procedure (15–17), and, to our knowledge, there are limited data on its standardized use in preoperative percutaneous CBT embolization (9).

## 5. Conclusion

Preoperative direct percutaneous embolization of CBT with Onyx is feasible, safe, and highly effective procedure, which achieves a high percentage of tumor devascularization and considerably facilitates the surgery by decreasing blood loss and operation time. Due to the relative rarity of these tumors, larger studies are challenging and future research is needed. The addition of BTO to this method provides surgeons with valuable information on potential intraoperative risks and necessary adjustments to the operation technique and limits for surgery.

## Data availability statement

The raw data supporting the conclusions of this article will be made available by the authors, without undue reservation.

## Ethics statement

Ethical review and approval was not required for the study on human participants in accordance with the local legislation and institutional requirements. Written informed consent for participation was not required for this study in accordance with the national legislation and the institutional requirements.

## Author contributions

MK: data collection and writing paper. JV: study design and supervision. BG, JR, MB, PC, and TK: data collection. All authors contributed to the article and approved the submitted version.

## Conflict of interest

The authors declare that the research was conducted in the absence of any commercial or financial relationships that could be construed as a potential conflict of interest.

## Publisher’s note

All claims expressed in this article are solely those of the authors and do not necessarily represent those of their affiliated organizations, or those of the publisher, the editors and the reviewers. Any product that may be evaluated in this article, or claim that may be made by its manufacturer, is not guaranteed or endorsed by the publisher.
